# Testing of Motor Coordination in Degenerative Neurological Diseases

**DOI:** 10.3390/healthcare10101948

**Published:** 2022-10-05

**Authors:** Maria Kalafati, Athanasios Kakarountas, Elisabeth Chroni

**Affiliations:** 1 Department of Computer Science and Biomedical Informatics, University of Thessaly, Papasiopoulou 2-4, 35131 Lamia, Greece; 2 Department of Medicine, University of Patras, 26504 Patras, Greece

**Keywords:** testing, motor coordination, screening tool

## Abstract

Parkinson’s disease (PD) is a progressive movement disorder caused by the death of dopamine-producing cells in the midbrain. PD is the most prevalent movement disorder of the central nervous system and affects more than 6.3 million people in the world. The changes in the motor functions of patients are not easy to be clearly and on-time observed by the clinicians and to make the most well-informed decisions for the treatment. The aim of this paper is the monitoring PD by designing, developing, and evaluating a prototype mobile App using a pressure pen, which collects quantitative and objective information about PD patients, thus allowing clinicians to understand better and make assumptions about the severity and the stage of Parkinson’s disease. This study presents a dynamic spiral test that can only be performed with tablet and pen pressure. Furthermore, the handwriting samples by PD patients and healthy controls individuals are collected by a computerized system, and the measurements of Spiral Deviation, Total Time, and Pen Pressure are processed. The results showed an accurate evaluation of the stage of Parkinson’s disease. Thus, the clinician may use the proposed PD telemonitoring system as a screening test, storing the history of all the patient’s measurements.

## 1. Introduction

Parkinson’s disease (PD) is a long- term degenerative disorder of the central nervous system that mainly affects the motor system. As the disease worsens, non-motor symptoms become more common. The symptoms usually emerge slowly. Early in the disease, the most obvious symptoms are shaking, rigidity, slowness of movement, and difficulty with walking. Thinking and behavioral problems may also occur. Dementia becomes common in the advanced stages of the disease. Depression and anxiety are also common, occurring in more than a third of people with PD. Other symptoms include sensory, sleep and emotional problems. The main motor symptoms are collectively called “parkinsonism”, or a “parkinsonian syndrome”. The cause of Parkinson’s disease is unknown but is believed to involve both genetic and environmental factors. The motor symptoms of the disease result from the death of cells in the substantia nigra, a region of the midbrain. The results are not enough dopamine in this region of the brain. The cause of this cell death is poorly understood, but it involves the build-up of proteins into Lewy bodies in the neurons. Diagnosis of typical cases are mainly based on symptoms, with tests such as neuroimaging used to rule out other diseases. There is no cure for Parkinson’s disease. Treatment aims to improve the symptoms. Initial treatment is typically with the antiparkinson medication levodopa (L-DOPA), followed by dopamine agonists when levodopa becomes less effective [1]. As the disease progresses and neurons continue to be lost, these medications become less effective while at the same time they produce a complication marked by involuntary writhing movements. Evidence for treatments for the non-movement-related symptoms of PD, such as sleep disturbances and emotional problems, is less strong [2]. In 2015, PD affected 6.2 million people and resulted in about 117,400 deaths globally. Parkinson’s disease typically occurs in people over the age of 60, of whom about one percent are affected. Males are more often affected than females at a ratio of around 3:2.

Today, many researchers have turned their attention to upgrading the medical approach to understanding the stage and the severity of PD via medical equipment or introducing innovative extra examinations, except from CT tomography, biomarkers, and other blood tests. In fact, the research “A novel computer-based technique for the assessment of tremor in Parkinson’s disease” is focused on the suitability and clinical value of a low-cost computer-based system as an aid to the diagnosis of PD, in particular the presence of tremor. All participants (12 patients and 10 controls) performed a shape-tracing task using a graphic tablet attached to a laptop. To assess the presence of tremors in the collected data, a statistical spectral analysis of the moment-to-moment fluctuations in the position signal of the output from the digitizing tablet was performed. This allowed the comparison of power spectrums obtained from the control and patient responses respectively. A peak in log power between the 5 Hz and 6 Hz can clearly be identified in the patient’s spectrum and is indicative of Parkinson’s related tremor and no similar peak could be seen in the control’s spectrum, suggesting this type of sequential task and automated data analysis may be useful in the diagnosis of tremor [3].

Moreover, Muhammed Erdem Isenkul et al. [4] proposed an alternative solution to the traditional method of paper and pencil of Spiral Static Test drawings (SST), which can be replaced by Dynamic Spiral Test (DST) that is realized on a tablet. They collected handwriting samples of patients who have been admitted to the Department of Neurology in Cerrahpaşa Faculty of Medicine, Istanbul University via a graphics tablet and the researchers compared SST and DST drawings of PD patients and healthy control subjects. The analysis demonstrates that the acceleration of SST is statistically closer to that of DST for control subjects when compared to the PD patients. It can be concluded that the SST and DST tests can be applied together in order to measure the cortical and motor performance of the subjects and can find use in diagnosis and telemonitoring applications of PD and some other similar neuropathological conditions.

Poonam Zham at al. [5] has proposed the use of the Composite Index of Speed and Pen-pressure (CISP) of sketching as for analyzing the severity level (SL) of PD. The participants drew an Archimedean spiral and speed, pen-pressure, and CISP were measured and analyzed to obtain their correlation with the severity of the disease. The correlation of speed, pen-pressure, and CISP with the severity of PD was −0.415, −0.584, and −0.641, respectively. The Mann–Whitney U test confirmed that CISP was suitable to distinguish between PD patients and healthy subjects, while the non-parametric k-sample Kruskal–Wallis test confirmed that it was significantly different for PD SL-1 and PD S-3. This shows that CISP during spiral sketching may be used to differentiate between CG and PD and between PD SL-1 and PD SL-3 but not SL-2.

Somayeh Aghanavesi et al. studied the measurement of temporal irregularity score (TIS) for patients at different stages of PD during each medication time points [6]. Both PD patients and healthy controls participated in the survey and the spiral tests on a smartphone were investigated before a single levodopa dose and at specific time intervals after the dose. Three movement disorder specialists rated videos of the patients based on UPDRS and the Dyskinesia scale. The differences in mean TIS between patients and healthy controls were estimated and there were proven when PD patients were in an advanced stage as well the capacity of TIS to detect changes from baseline (before medication) to later time points was assessed. TIS had good test-retest reliability and it was responsive to single-dose levodopa treatment. Since TIS is an upper limb high-frequency-based measure, it cannot be detected during clinical assessment.

Manuel Gil-Martín et al. [7] proposed the use of analyzing a convolutional neural network (CNN) for PD detection from drawing movements, which combines the feature extraction (convolutional layers) and classification (fully connected layers). The CNN has inputs which are the module of the Fast Fourier transform in the range of frequencies between 0 Hz to 25 Hz. Using the public dataset: Parkinson Disease Spiral Drawings Using Digitized Graphics Tablet dataset, they analyzed into X and Y directions the discrimination capability during drawing movements of individuals. The accuracy of this work is 96.5%, a F1-score of 97.7%, and an area under the curve of 99.2%.

Iqra Kamran et al. presented an approach of patients’ handwriting samples for early diagnosis of PD [8]. They include different Parkinson’s datasets, which are PaHaW dataset, HandPD dataset, NewHandPD dataset, and Parkinson’s Drawing Datasetand, and applied deep transfer learning algorithms to overcome the challenge of high variability in the handwritten material. In their analysis, they evaluated six main transfer learning architectures, namely AlexNet, GoogleNet, VGGNet-16/19, and ResNet-50/101. They succeed in excellent PD identification performance with 99.22% accuracy on the illuminated tasks of combined HandPD, NewHandPD, and Parkinson’s Drawing datasets, demonstrating the superiority of our approach over current state-of-the-art methods.

Elina Kuosmanen et al. [9] proposed a work related to this work, which describes the implementation of the digitized version of the spiral drawing test for Android devices. In this application, they have introduced both the spiral test and the square-shape drawing and in the survey eight PD patients and six healthy controls participated and the error rate and the drawing speed were measured. The results of the trials were a clearly different accuracy between the PD patient and the healthy individuals between the two drawing tasks.

Besides focusing only on Parkinson’s disease, Andrius Lauraitis et al. [10] presented a new approach which addressed other degenerative disorders of the central nervous system, such as Huntington’s disease, Alzheimer’s Disease, mild cognitive impairment, and dementia. They proposed a smart application, called “Neural Impairment Test Suite” (NITS), for Android smartphones and tablets which concerns the self-administered cognitive testing (SAGE) methodology that used finger tapping and voice features acquired from the sensors of the device. The experiments were realized in patients with neurological disorders (one with Parkinson’s disease, three with Huntington’s disease, one with early dementia, one with cerebral palsy, one post-stroke) and eight healthy controls. The data are collected in an Android device and measure cognitive, hand tremor, energy expenditure, and speech features of subjects. According to the statistical analysis, they used 13 classifiers for combined finger tapping and SAGE features, and 96.12% accuracy was achieved and using bidirectional long short-term memory (BiLSTM) (94.29% accuracy) for speech analysis features.

Benjamin I Ferleger et al. [11] proposed a pilot study about a tablet and a mobile-based application for remote diagnosis and analysis of movement disorder symptoms. More specifically, in this application, the patients are called to follow, with a pen, a drawing task, especially with the spiral and line-drawing tasks of the Fahn–Tolosa–Marin tremor rating scale serving as the task in this survey. The data are collected in a cloud, which is analyzed quantitatively, and drawing smoothness, pressure applied, and other measures are estimated. The maximum cross-validated classification accuracy on a preliminary sample set was 98.3%.

Except for applications that specialize only in spiral drawings, Hung N. Pham et al. [12] introduces a novel study that incorporates voice and spiral drawing for better detection of PD severity level. In their study, they use various machine learning models and succeed with a great accuracy level for PD recognition. Using pairwise correlation and k-means clustering techniques the highest accuracy of 95.89% is obtained using an ensemble of 3 classification models. The best accuracy of 99.6% is achieved using the k-Nearest Neighbors classifier in the Dynamic Spiral Test (DST) and accuracy of 98.8% and 94.9% are achieved using the Logistic Regression classifier and the Adaptive Boosting classifier on the Static Spiral Test (SST) and Stability Test on Certain Point (STCP). Finally, the trials were implemented into a touch-enabled smartphone-based application.

The objective of this study is to design, develop, and evaluate a prototype digital application for mobile appliances and tablets using a pressure pen, which collects quantitative and objective information about the PD patients’ handwriting dexterities, thus allowing clinicians to monitor the deterioration of motion or response to treatment and to make assumptions about the prognosis severity and the stage of Parkinson’s disease. A cardinal sign of PD is the lack of coordination of fine movements of the hand for a common procedure such as writing or drawing. Clinicians often offer a piece of paper to the patient and ask them to draw geometric shapes in order to decide the effect of the disease on this function. A digital tool, easy to be used in routine visits (and even at home), characterized for accuracy and reproducibility, would be a valuable asset to neurologists and caregivers.

## 2. Materials and Methods

### 2.1. Description of the Mobile App

As described in Section 1, the aim of this study is to design a novel mobile application, which will be a useful tool to the medical society and will be effective in the diagnosis and monitoring of Parkinson’s disease. The main novelty of the presented tool is that it may be used for generating real-time data on the writing ability of the patient and creating datasets in historical order. The data are then read by the neurologist and the evolution of the PD is assessed. In contrast to other works adopting the spiral test, the digital application is offering an accurate and timely assessment of the PD patient status. The use of the application by the PD user may occur in any environment, apart from the clinic, with the assistance of the caregiver.

For the evaluation of the application, it was installed on a tablet. Then, the handwriting dataset was constructed using a pressure pen as the input device, for recording the movement and the applied pressure. Specifically, the tablet is connected with the Sonar Pen, which is a pressure pen that estimates the pressure or the applied force by the patient on the screen of the tablet. Unlike the traditional tests, using a pencil and paper, the patient’s digitized handwriting gives valuable digital features which are accurate x-y-z coordinates, the precise pressure applied to the screen, the pen grip angle, and the total time that the patient required to complete the drawing task.

**Figure 1:**
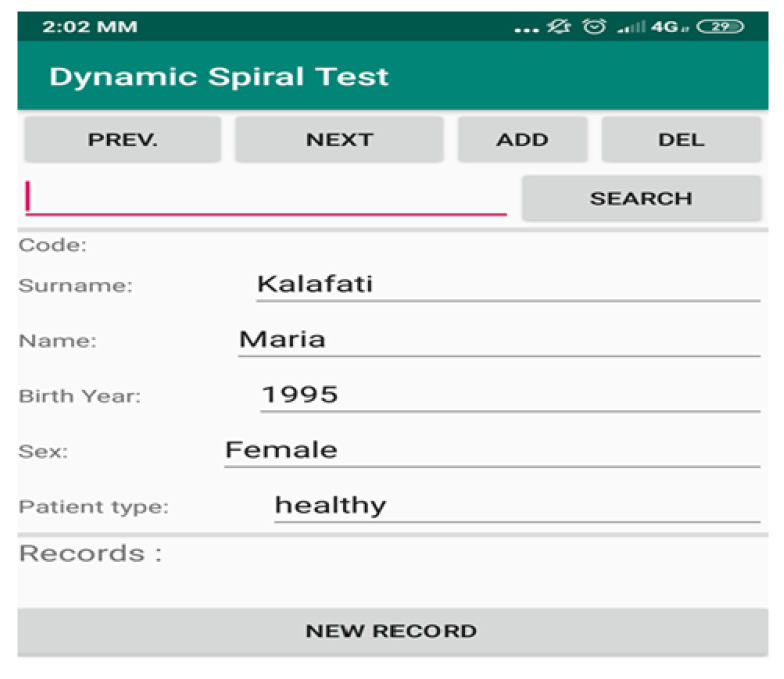
The initial screen of the Dynamic Spiral Test.

The procedure to initiate the test and capture the results is simple and is described in the following. Initially, the clinician launches the app, which is found in the installed apps of the mobile or tablet, and fills in the fields with the required information, such as “surname”, “name”, “birth year”, “sex” and “patient type”, as seen in Figure 1. In the field “patient type” the clinician indicates if the Parkinson’s test is realized in a healthy person or a PD patient. The application, also, gives in every addition a unique “code number”, which is incremental, to indicate the new registered patient/user. Selecting the option “ADD” the addition of a new patient is confirmed. The clinician then has the option to search for any registered patient, by surname or code number, and access the previous tests and assessments associated with the particular patient. There is also the option to delete an addition using the “DEL” option. The last option available to the clinician is to perform a new test, that is recording new data regarding the handwriting movement of the user, by selecting the option “NEW RECORD”, which brings to the forefront the spiral test screen.

**Figure 2:**
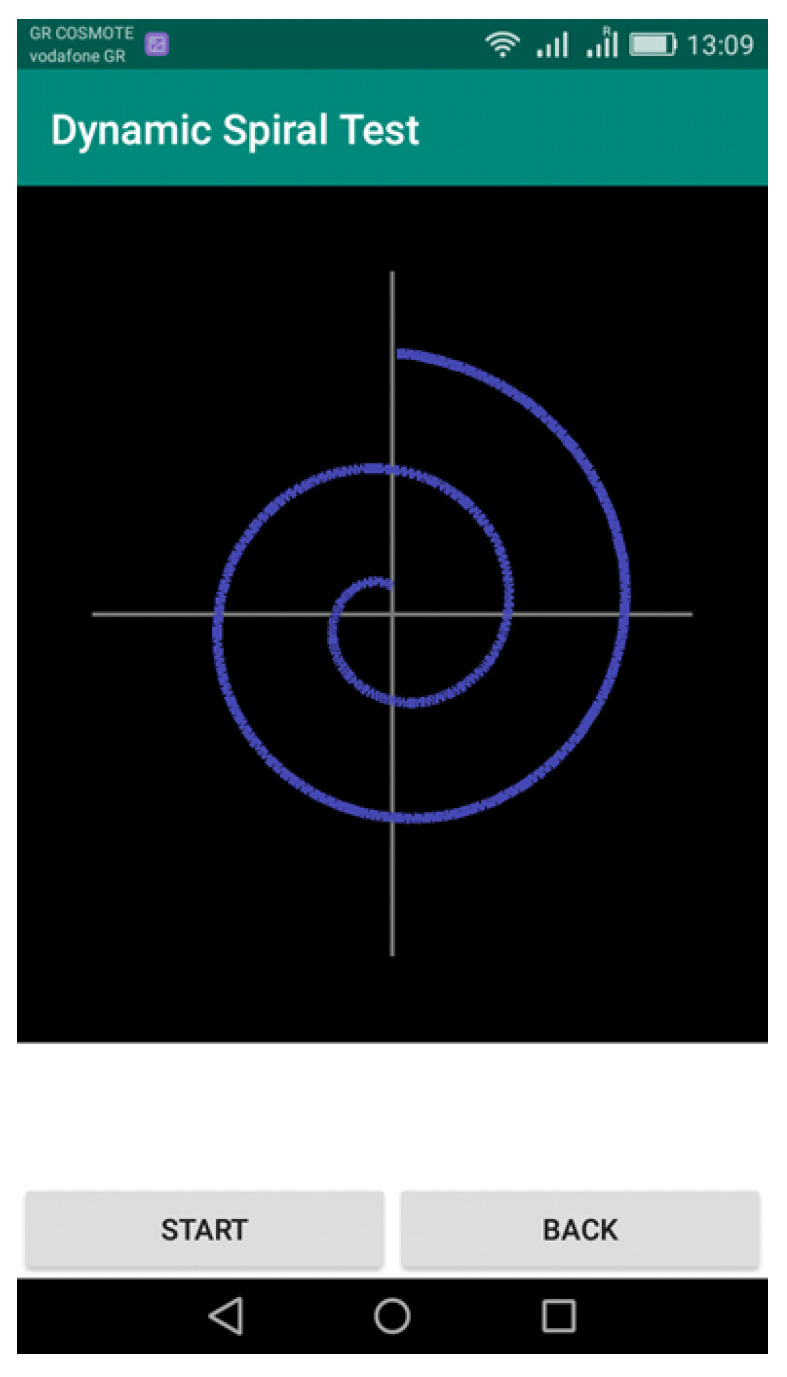
Second screen of Dynamic Spiral Test—the spiral drawing task.

In the spiral drawing task screen, the clinician initiates the test by selecting the “START” option. Then the patient, using the pressure pen, follows the spiral line trying to draw accurately the spiral shape. The movement is inwards out, as seen in Figure 2.

**Figure 3:**
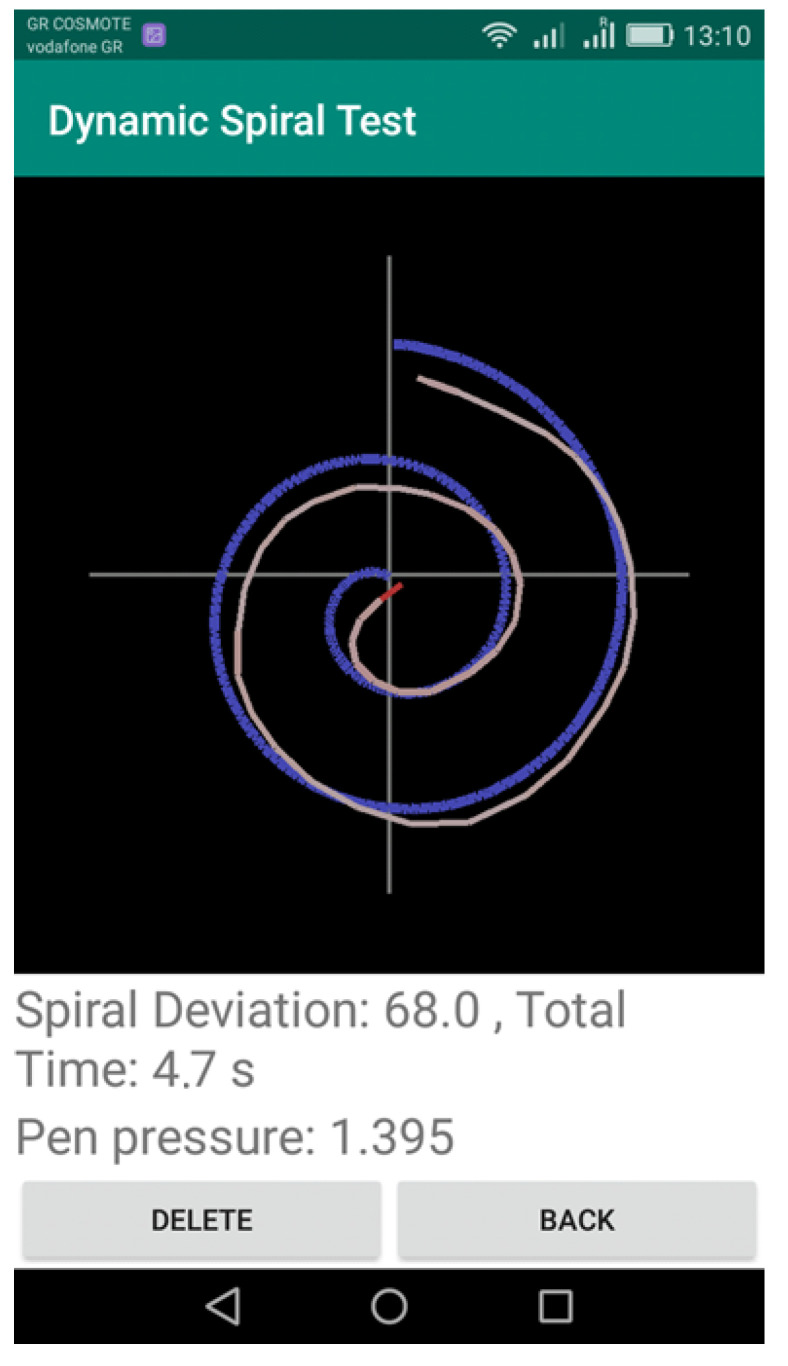
Screenshot of the application showing the spiral drawn by the patient using the SonarPen.

When the patient completes the spiral test, the clinician selects the “STOP” option to indicate the completion of the test. Then, the drawn shape is depicted over the initial spiral shape, to indicate the deviations in the drawing and also the pressure (red for high pressure and shades of red for low pressure), as shown in Figure 3.

The Dynamic Spiral Test application offers three different assessments of the completed task:

- The measurement “Spiral Deviation” is the ratio of the area between two lines of the spiral test to the screen size multiplied by 10,000. With this value, the clinician can draw the right conclusions about the ability of the patient to lead the line of the spiral. More specifically, the malfunction of the fingers is observed due to tremor and in combination with the above two measurements, the symptoms of bradykinesia, tremor, and malfunction are examined by the clinician.
- The measurement “Time” relates to the total time needed by the patient to complete the spiral test. A long time for a patient to complete the task is directly associated with the stage of the PD disease, as described in previously mentioned works.
- The calculation “Pen Pressure” relates to the exercised pressure by the patient. If the pressure is greater than the expected one (as derived by tests by healthy users), then the line is more intense, red, and thicker, whereas if the exercised pressure is weak then the color of the line approaches the pink color, as in the Figure above. If the patient has tremor at a great scale, the exercised pressure, which is expected by the pen on the tablet will be weaker, so the patient will try to increase the pressure to achieve the stability of the pen.

Thus, the suggested three values of the application offer an indication of the severity of Parkinson’s disease.

### 2.2. Application Program Design

The proposed application was developed targeting Android-based smartphones and tablets. It was developed in Java with Android Studio v3.52 for Linux, using an object-oriented methodology.

The application contains five main parts:

- The first part handles the graphic user interface (GUI). It uses Android SDK functions and consists of two screens, the one that shows the patient’s data and the other the spiral sketch graph (API level 29). This approach is the most popular and common way for Android App development, supported by Google Inc., Mountain View, CA, USA.
- The second part is responsible for the calculation of the surface between the curve drawn by the patient and the spiral displayed on the screen (Spiral Deviation). A special algorithm is used for this. For each point of the curve drawn by the patient (curve with red color in screenshot) the application stores the following values: x position, y position, time in milliseconds, pressure on this point, and its spiral angle. The Spiral angle is a value that starts with the value of 90 (the polar angle of the first point) and it is increased by 720 degrees (two full circles up to the final point). In this way, each point of the patient’s curve is related to a point of the test spiral. The calculation of the spiral deviation uses the distance of these two points.
- The third part is responsible for acquiring and displaying the relative pressure values of the Sonar pen as sampled from the Tablet’s microphone input.
- A fourth part is aimed to generate a dedicated audio signal/tone, which is forward to the stereo audio output (L-R) of the Tablet. This signal is used to implement the battery-less pressure sensing of the Sonar Pen.
- Finally, a fifth part is used to store and fetch data from internal storage.

### 2.3. The Pressure Pen

**Figure 4:**
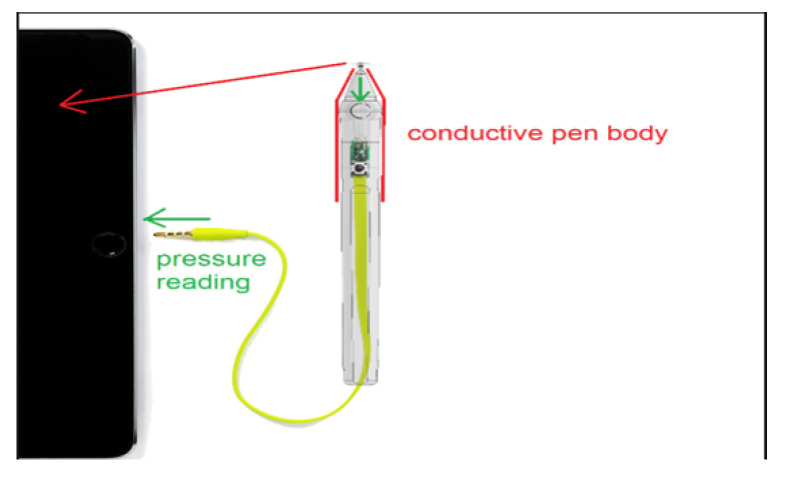
Connection between the Sonarpen and the Tablet.

The pressure pen selected for the application is the Sonar pen. The Sonar pen is a smart stylus, which is used for digital drawing and has all the standard features (e.g., position, angle, and pressure capturing), at a relatively low cost (compared to other similar devices). The de facto standard of a smart pen is to offer the following functions (if not all): pressure sensing, palm rejection, and shortcut button. The technology of the Sonar pen is based on the earphone solution, which eliminates the costs of expensive electronics, controlling circuits, Bluetooth, and rechargeable batteries and replaced them with a circuit that communicates with the tablet through the standard audio channel. To detect the pressure, the Tablet sends a dedicated audio signal waveform to the stereo audio output (L-R), the signal waveform passes through a simple voltage divider circuit using a force-sensitive resistor which changes according to the pressure applied to it, such in Figure 4. The modulated waveform is then sent back to the Tablet using the microphone input, for reading the voltage drop over the force-sensitive resistor which relates to the applied pressure.

### 2.4. Assessment of the Test Procedure for the PD Patients

**Figure 5:**
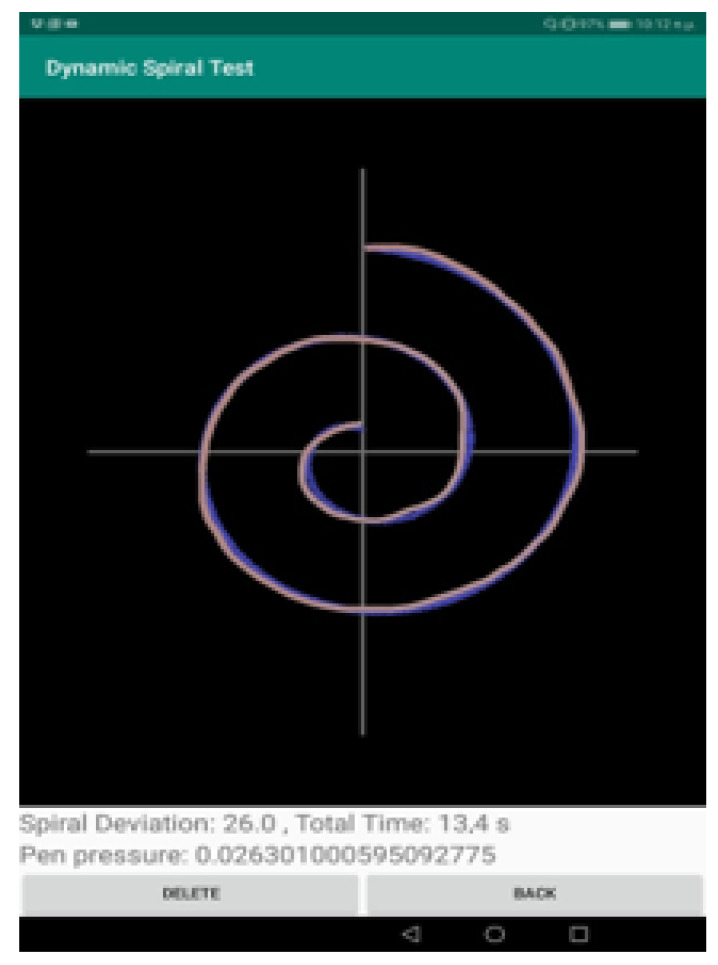
Spiral Tests of Healthy Control.

**Figure 6:**
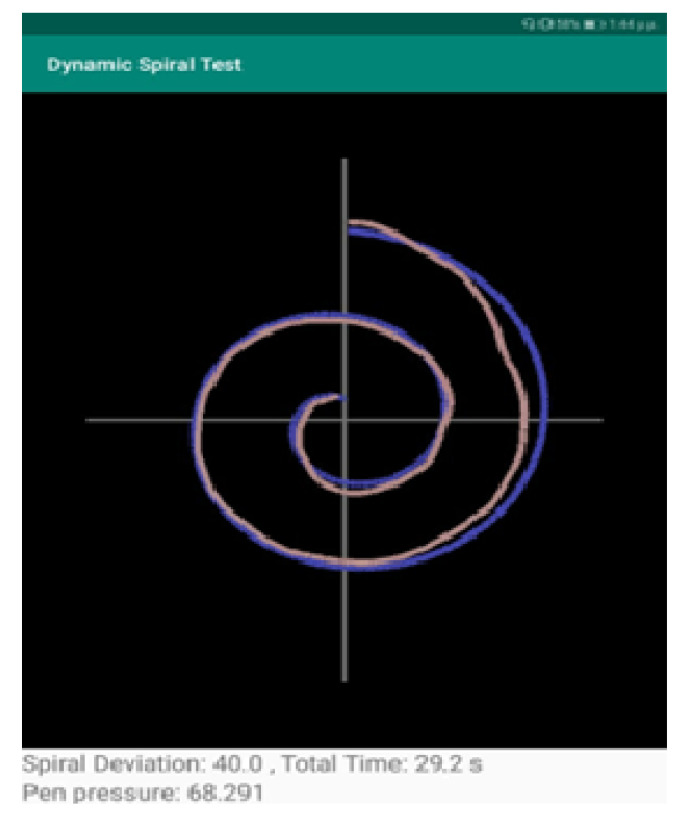
Spiral Tests of PD Patient.

The tests took place at the University General Hospital of Patras “Panagia h Bohthia” at the Neurological Clinic with the guidance of Dr Zinovia Kefallopoulou, Neurologist, and Dr. Elisabeth Chroni, Neurology Professor of the Department of Medicine. The tests were realized by PD patients and a healthy control group. The patients signed a detailed informed consent form before participating in the tests and they had the opportunity of rejection. The form assured participants of medical confidentiality and concealment of their personal information. The patients who participated in the tests were selected with two criteria. The first is that all the PD patients were in the same severity stage of the disease. It was complicated to discriminate among all the patients of the clinic the patients who presented the same behavior and characteristics, limiting the patients, however, to adequate sample size. In addition, the second criterion was that the patients follow the same medication for a quite long time. It is important to be noticed that all the patients suffered from PD for over a decade. Twelve PD patients and twelve healthy controls participated in the tests. For PD patients, there were 4 women, 2 in the decade of 40–50 and 2 in the decade of 50–60, and 8 men, 4 in the decade of 40–50 and 4 in the decade of 50–60, and for healthy controls, there were 6 women, 3 in the decade of 40–50 and 3 in the decade of 50–60 and 6 men, 3 in the decade of 40–50 and 3 in the decade of 50–60, were recruited in repeated spiral drawings of the application using the tablet with the Sonarpen. The tablet has 9.6 inches touch screen with a screen resolution of 1280 × 800 and recorded both positions (x and y coordinates). No participant in the study had cognitive or visual problems to the extent to which it could influence their test performance. Firstly, the clinician was noticing the personal information of the patient or the individual from the healthy control group. The subjects were informed by the clinician how to draw the spiral test and afterward, they performed the test. They were seated on a chair and performed the tests using the pressure pen with the tablet, which was on a stable spot. All data collected was then stored in a file of the application in the device’s storage memory. Figure 5 and Figure 6 are examples of dynamic spiral tests of healthy controls and PD patients respectively.

Statistical analysis was performed using the parametric statistical procedure T-test. In the study, T-test hypothesis tests were performed for statistical analysis of the parameters of independent samples in the following different cases. In each different case mean, Standard Deviation, and Standard Error Mean were observed and their values are placed in each table separately. Moreover, Levene’s test is also used in the study, which is an inferential statistic used to assess the equality of variances for a variable calculated for two or more groups.

## 3. Results

- In Table 1, which is organized as follows, the independent variable is HEALTH (indicating whether the tests were performed by PD patients or healthy individuals) and dependent values are Standard Deviation (SD in %), Total Time (TT in sec), and Pen Pressure (PP as a number corresponding to the sum of the pixels multiplied by the difference of the force applied force (Newton—N) to the typical pen pressure (1.4–1.5 N)—the bigger the number the higher the pressure in many points, while typical handwriting pressure tends to zero) separately:

- In Table 2, for PD patients the mean value of SD was 33.175 and for healthy was 29.833. As expected, PD patients present a greater deviation in SD value in drawing test, as result of the tremor and difficulty in moving of the hand of the disease. Additionally, the mean value of the TT variable, for PD patients, is 20.13 while for healthy it is 12.73. The total time to complete the spiral test is quite longer in the patient, as opposed to the healthy controls due to bradykinesia and other motor symptoms of Parkinson’s disease. In PD patients the mean PP is 47,696.11 and for healthy it is 0.02. Because of the motor symptoms of the disease, PD patients exercise significantly greater pressure in their attempt to complete the dynamic spiral test in comparison with the healthy controls, who have no difficulties.
  **For SD**: Testing for comparison of sample variances: Levene’s Test for Equality of Variances (H0: variances do not differ). From the importance of this control Sig. = 0.286 > 0.05 we conclude that there is no significant difference in variances and therefore we conclude that variances do not differ. The significance of the test is Sig. = 0.211 > 0.05 we conclude that the variance SD does not depend from the health. The results are not statically significant. Thus, we infer that both of healthy controls and patients present spiral deviation of the dynamic spiral test of our application. From our sample it follows that the SD does not depend on whether you are healthy or PD patient.
  **For TT**: Testing for comparison of sample variances: Levene’s Test for Equality of Variances (H0: variances do not differ). From the importance of this control Sig. = 0.235 > 0.05 we conclude that there is no significant difference in variances and therefore we conclude that variances do not differ. Because the significance of the test is Sig. = 0.008 < 0.05 we reject the null hypothesis and conclude that the total time depend from the health and there is a statistically significant difference in the value of TT between patients and healthy. Indeed, once again we confirm that patients need more time to complete the dynamic spiral test in relation with healthy subjects.
  **For PP**: Testing for comparison of sample variances: Levene’s Test for Equality of Variances (H0: variances do not differ). From the importance of this control Sig. = 0.00 < 0.05 we conclude that there is a significant difference in variances and therefore we conclude that variances differ. From the statistical analysis we observe that the the significance of the test is Sig = 0.00 < 0.05, so the pen pressure depends from whether is healthy control or PD patient. Actually, there is a statistical difference between healthy and patients regarding the variable PP.

- In Table 3, only PD patients are analyzed and the age is considered as the independent variable, while SD, TT, PP are dependent variables (separately).

- The results for the age variable are summarized in Table 4: For people aged 50–60 years, the mean SD is 37.87 while for 40–50 years it is 28.48. Here, we can conclude that the age affects the SD of the patients and is probably due to the fact as the age group increases, the tremor and the other motor symptoms, such as instability, are increased too.
  For people aged 50–60 years, the mean of the TT variable is 20.82 while for the 40–50 years old it is 19.43. We notice that the longer a patient has been affected by the disease, despite being under the influence of medication, the more complicated it is to complete the test, and therefore it takes longer to complete the dynamic spiral test.
  For people aged 50–60 years, the average of the PP variable is 59,088.37 while for 40–50 years it is 36,303.85. As it is mentioned above, and at this point we observe that the greater age group needs more pressure to performing the dynamic spiral test. We come to the conclusion that although medication reduces and exacerbates the symptoms of the disease, it appears from our analysis that over time all three variables are affected. **For SD**: Testing for comparison of sample variances: Levene’s Test for Equality of Variances (H0: variances do not differ). From the importance of this control Sig. = 0.740 > 0.05 we conclude that there is no significant difference in variances and therefore we conclude that variances do not differ. The significance of the test is Sig. = 0.015 < 0.05, thus we conclude that the variance of SD depends from the age of patients there is a statistically significant difference in the value of SD between the patient group of 50–60 years and patient group of age between 40–50. Actually, as the age of the patients increases, we notice that the value of the spiral deviation increases too.
  **For TT**: Testing for comparison of sample variances: Levene’s Test for Equality of Variances (H0: variances do not differ). From the importance of this control Sig. = 0.511 > 0.05 we conclude that there is no significant difference in variances and therefore we conclude that variances do not differ. The significance of the test is Sig. = 0.753 > 0.05 and we make the conclusion that there is no statistically significant difference in the value of TT between 50-60 years and 40-50. The SD variance does not depend from the age of the patient.
  **For PP**: Testing for comparison of sample variances: Levene’s Test for Equality of Variances (H0: variances do not differ). From the importance of this control Sig. = 0.281 > 0.05 we conclude that there is no significant difference in variances and therefore we conclude that variances do not differ. The significance of the test is Sig. = 0.146 > 0.05, so we conclude that the PP variance does not depend from the age group of patients.

- In Table 5, only PD patients are analyzed and the sex is considered as the independent variable, while SD, TT, PP are dependent variables.

- The results for the sex variable are summarized in Table 6: For women, the mean of SD is 35.28 while for men it is 32.13. The women present a little greater SD in comparison with men.
  For women, the mean of TT is 18.33 while for men it is 21.03.
  For women, the average PP is 45,035.05 while for men it is 49,026.64.
  From these three valuable variables emerge the conclusion that the symptoms of the disease affect a greater percentage of women compared to men. However, in women, the development of symptomatic PD may be delayed by higher physiological striatal dopamine levels, possibly due to the activity of estrogens. This could explain the epidemiological observations of a lower incidence and higher age at onset in women. Women also presented more often with tremor which, in turn, is associated with milder motor deterioration and striatal degeneration. Taken together, these findings suggest a more benign phenotype in women with PD, according to [13]. So, combining these results with our conclusions, the treatment of Parkinson’s disease which is focused on oestrogens works more effectively on symptoms in men than in women although the disease occurs more in the male population.
  **For SD**: Testing for comparison of sample variances: Levene’s Test for Equality of Variances (H0: variances do not differ). From the importance of this control Sig. = 0.138 > 0.05 we conclude that there is no significant difference in variances and therefore we conclude that variances do not differ. The significance of the control is Sig. = 0.503 > 0.05 we conclude that there is no statistically significant difference in the value of SD between women patients and men patients. **For TT**: Testing for comparison of sample variances: Levene’s Test for Equality of Variances (H0: variances do not differ). From the importance of this control Sig. = 0.807 > 0.05 we conclude that there is no significant difference in variances and therefore we conclude that variances do not differ. The significance of the control is Sig. = 0.561 > 0.05 we conclude that there is no statistically significant difference in the value of TT between women and men in our sample. The value of TT does not depend from the sex of patients.
  **For PP**: Testing for comparison of sample variances: Levene’s Test for Equality of Variances (H0: variances do not differ). From the importance of this control Sig. = 0.799 > 0.05 we conclude that there is no significant difference in variances and therefore we conclude that variances do not differ. The significance of the control is Sig. = 0.820 > 0.05, so we conclude that there is no statistically significant difference in the value of PP between women and men in our sample.

- In Table 7, only healthy individuals are analyzed and the age is considered as the independent variable, while SD, TT, PP are dependent variables.

- The results for the age variable are summarized in Table 8:
  For people aged 50–60 years, the mean of SD is 30.00 while for 40–50 years it is 29.67.
  For people aged 50–60 years, the mean of the TT variable is 12.72 while for the 40–50 years old it is 12.73.
  For individuals aged 50-60 years, the mean of the PP variable is 0.01943 while for the age group 40–50 it is 0.1869.
  Comparing, the tables of the second case with the present case, where in both the age is the analyzing variable, we observe that exist an important difference in SD, TT and PP measurements, as we expected. Although the patients are under their medication for a long time, there is exist difficulties due to motor symptoms.
  **For SD**: Testing for comparison of sample variances: Levene’s Test for Equality of Variances (H0: variances do not differ). From the importance of this control Sig. = 0.277 > 0.05 we conclude that there is no significant difference in variances and therefore we conclude that variances do not differ. The importance of control is Sig. = 0.920 > 0.05. We make the conclusion that the SD variance does not depend from the age group of 40–50 and 50–60.
  **For TT**: Testing for comparison of sample variances: Levene’s Test for Equality of Variances (H0: variances do not differ). From the importance of this control Sig. = 0.219 > 0.05 we conclude that there is no significant difference in variances and therefore we conclude that variances do not differ. The significance of the test is Sig. = 0.996 > 0.05, so we conclude that there is no statistically significant difference in the value of TT between 50–60 years and 40–50.
  **For PP**: Testing for comparison of sample variances: Levene’s Test for Equality of Variances (H0: variances do not differ). From the importance of this control Sig. = 0.686 > 0.05 we conclude that there is no significant difference in variances and therefore we conclude that variances do not differ. The significance of the control is Sig. = 0.92 > 0.05 and we conclude that there is no statistically significant difference in PP value between 50–60 years and 40–50. The value of PP is not affected from the age group on healthy controls.

- In Table 9, only Healthy individuals are analyzed and the sex is considered as the independent variable, while SD, TT, PP are dependent variables.

- The results for the sex variable are summarized in Table 10:
  For women, the mean of SD is 30.50 while for men it is 29.17.
  For women, the mean of the TT variable is 13.20 while for men it is 12.25.
  For women, the mean of PP is 0.0214 while for men it is 0.0167.
  We observe that there is no significant difference between healthy men and women
  **For SD**: Testing for comparison of sample variances: Levene’s Test for Equality of Variances (H0: variances do not differ). From the importance of this control Sig. = 0.143 > 0.05 we conclude that there is no significant difference in variances and therefore we conclude that variances do not differ. The significance of the test is Sig. = 0.685 > 0.05 we conclude that there is no statistically significant difference in the value of SD between women and men. The SD variance does not depend from the sex of healthy controls.
  **For TT**: Testing for comparison of sample variances: Levene’s Test for Equality of Variances (H0: variances do not differ). From the importance of this control Sig. = 0.763 > 0.05 we conclude that there is no significant difference in variances and therefore we conclude that variances do not differ. The significance of the control is Sig. = 0.766 > 0.05 we conclude that there is no statistically significant difference in the value of TT between women and men. Thus, the TT value between women and men remains unaffected.
  **For PP**: Testing for comparison of sample variances: Levene’s Test for Equality of Variances (H0: variances do not differ). From the importance of this control Sig. = 0.323 > 0.05 we conclude that there is no significant difference in variances and therefore we conclude that variances do not differ. According the significance of the control is Sig. = 0.371 > 0.05, so we conclude that there is no statistically significant difference in the value of PP between women and men.

### Visualization of the Statistical Analysis

**Figure 7:**
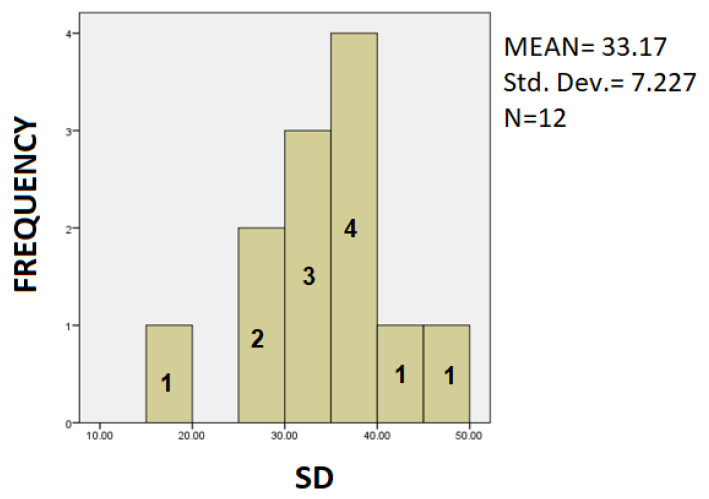
Barchart showing the frequency of the PD patients and the clustering of SD values.

**Figure 8:**
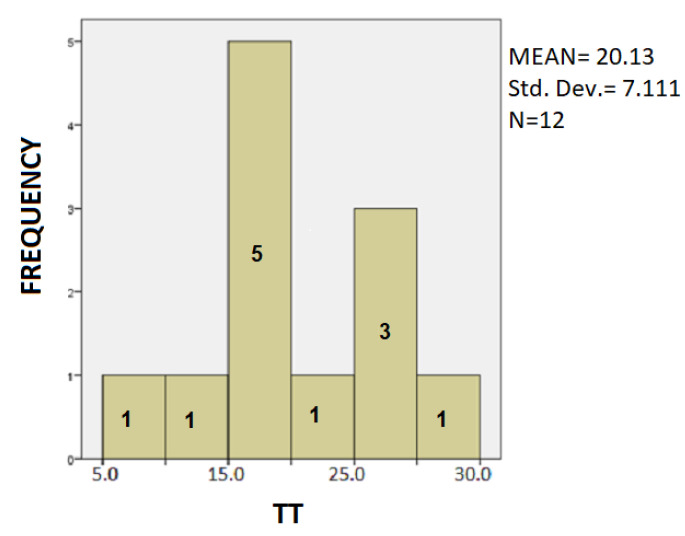
Barchart showing the frequency of the PD patients and the clustering of TT values.

**Figure 9:**
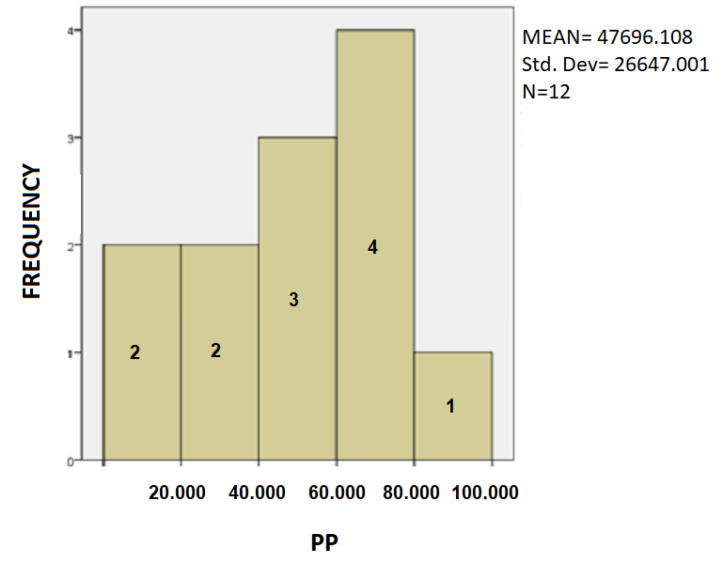
Barchart showing the frequency of the PD patients and the clustering of PP values.

The following is a statistical analysis using bar charts. In Figure 7, Figure 8 and Figure 9 the Patients’ Frequency bar charts for the three parameters are depicted.

In Figure 7 we observe that most patients of the sample present a value between 35–40 of Spiral Deviation measurement.

In Figure 8 we observe that most patients of our sample use a total time of 15–20 s for completion of the spiral test, whereas the mean value of the total time is greater, i.e., the most patient of our sample use less time than the total mean time of the group to complete the motive test.

In Figure 9 we observe that most patients achieve values between 60,000–80,000, but the total mean value of Pen Pressure measurement is the lowest, which means that most patients exercise more pressure on the pen to draw the spiral test.

**Figure 10:**
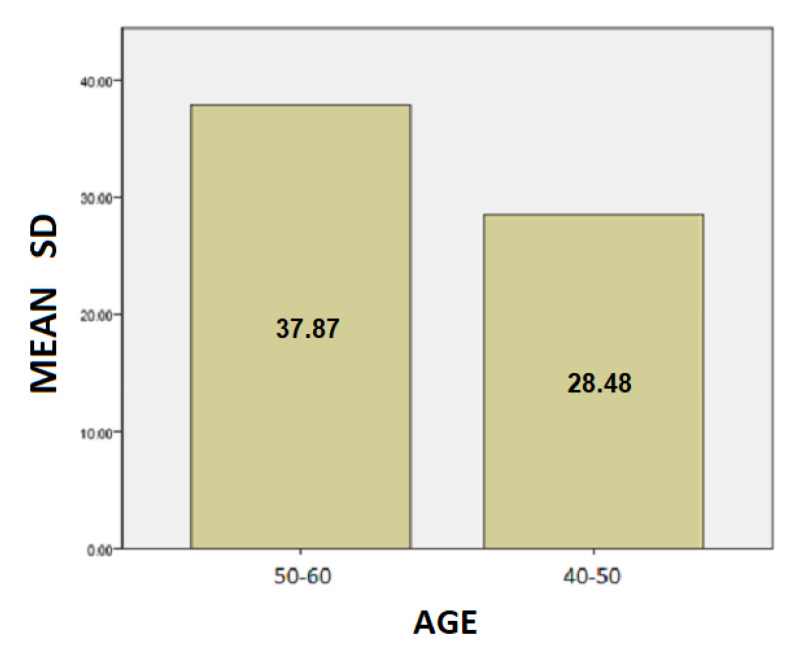
Barchart for patients with the mean value of SD by age.

**Figure 11:**
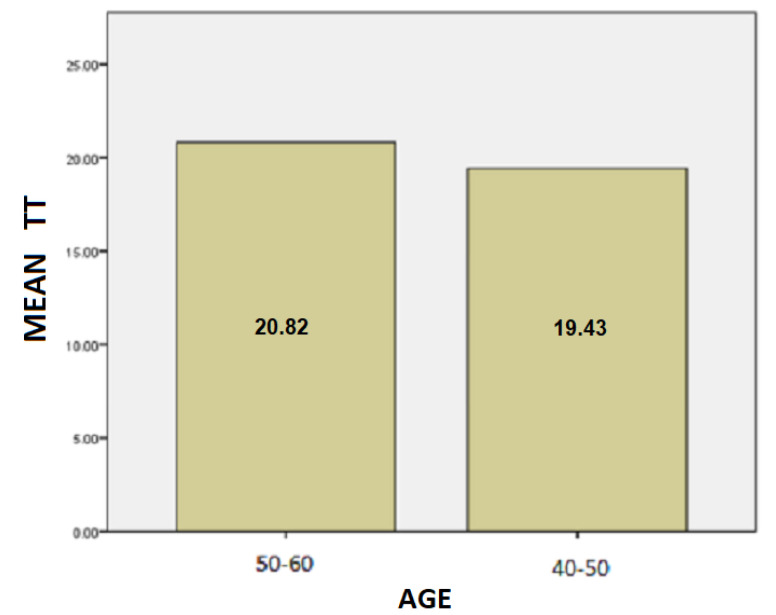
Barchart for patients with the mean value of TT by age.

**Figure 12:**
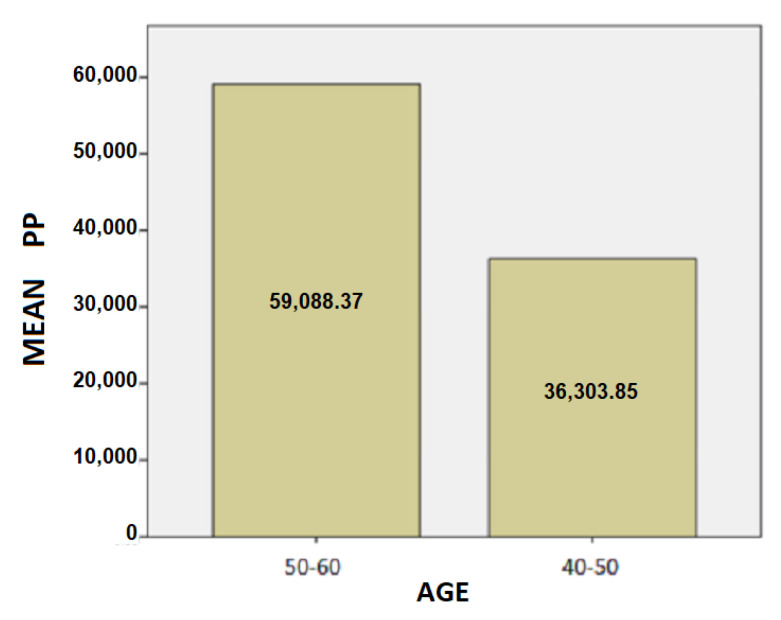
Barchart for patients with the mean value of PP by age.

Following are bar charts (as depicted in Figure 10, Figure 11 and Figure 12) for patients with the mean values of the variables depending on the age group.

## 4. Discussion and Conclusions

This study presents a novel tool for the quantitative estimation of movement disorders. The aim was to develop a flexible test, suitable for clinical practice that will allow monitor of certain muscle activities during the progress of the disease. It could also be useful for the assessment of drug or surgery treatment response. The preliminary application of the developed tool in several PD patients was successful. All patients as well as healthy controls followed the instructions and perform the test. No more than three repetitions were necessary in order to complete the task.

The main conclusion was that there is a statistically significant difference between healthy and PD patients for total time and pen pressure, reflecting the bradykinesia and rigidity of the patients respectively. Specifically, the poverty movement and the slow reaction to perform daily activities, which are characteristics of PD resulted in increased time spent to complete this specific task. Another characteristic of PD, i.e., increased muscle tone, known by the term rigidity was reflected by the enhanced pressure exerted on the pen. On the other hand, inter-group comparison of the spiral deviation did not reach a significant level, possibly because in PD voluntary activity is not predominately affected. The coordination of hand movements and is not deranged in PD, at least early on. Likewise, a tremor is often called a resting tremor, since it is not interfered with the intended motion as in an idiopathic senile tremor. It would be interesting to perform the same test in patients with idiopathic tremor or ataxia where it is expected to find severely abnormal values of spiral deviation.

The spiral deviation was higher in older patients, perhaps suggesting the greater disease severity. No other meaningful statistical results were obtained. In addition, one technical advantage of the application is the digitization and the automation of the results. In the traditional way, clinicians who are occupied with Parkinson’s disease, such as physiotherapists etc., could observe only the tremor of the patient with the observation of the distance between the patient’s line and the motive’s line. Now with the application, clinicians can observe automatically the exercised pressure of the patient on the tablet and the time that the patient needs to complete the test, except from the distance.

The main limitation of this study is the small sample size both for patients and healthy volunteers. It is possible that PD patients at a later stage of the disease demonstrate higher deviation from normality. The role of age should be examined in a future study as well. The next step would be the application of this test in a large sample of patients with extrapyramidal syndrome, as well as in patients with spasticity (pyramidal syndrome) or cerebellar ataxia in order to detect disease-specific differences.

The contribution of this work in relation to the other projects is not only the digitization of the quantitative measurements, such as the spiral deviation, the motive completion time, and the pressure that the patient exercise on the tablet with the pen, but this work is observing and analyze statically the differences between the age groups of patients and the differences between the sex of patients, each time different dependent and independent variable.

In brief, a novel custom-made software was developed in order to provide clinicians with a practical tool for the evaluation of movement disorders. Its preliminary application was successful allowing differentiation between PD and healthy subjects. Estimation of its specificity and sensitivity would require future studies in a large cohort of patients.

## Figures and Tables

**Table 1:** Group Statistics—HEALTH variable.

	HEALTH	N	MEAN	Std. Deviation	Std. Error Mean
SD	PATIENTS	12	33.1750	7.2267	2.0862
(% pixels)	HEALTHY	12	29.8333	5.3229	1.5366
TT	PATIENTS	12	20.1250	7.1106	2.0526
(sec)	HEALTHY	12	12.7250	5.1517	1.4872
PP	PATIENTS	12	47,696.1080	26,647.0019	7692.3269
(N * pixels)	HEALTHY	12	0.0191	0.0088	0.0026

**Table 2:** Independent Samples Test—HEALTH.

		Levene’s Test		T-Test for Equality of Means						
		for Equality								
		of Variances		95% Confidence Interval of the Difference						
							**Mean**	**Std. Error**		
		**F**	**Siq.**	**t**	**df**	**Sig.** ^1^	**Difference**	**Difference**	**Lower**	**Upper**
SD	Equal variances assumed	1.195	0.286	1.290	22.000	0.211	3.3416	2.5909	−2.0317	8.7150
	Equal variances not assumed			1.290	20.221	0.212	3.3416	2.5909	−2.0592	8.7426
TT	Equal variances assumed	1.490	0.235	2.919	22.000	0.008	7.4000	2.5347	2.1432	12.6567
	Equal variances not assumed			2.919	20.054	0.008	7.4000	2.5347	2.1134	12.6865
PP	Equal variances assumed	28.269	0.000	6.200	22.000	0.000	47,696.0892	7692.3268	31,743.1797	63,648.9987
	Equal variances not assumed			6.200	11.000	0.000	47,696.0892	7692.3268	30,765.3919	64,626.7865

^1^ Sig. (2-tailed).

**Table 3:** Group Statistics—PD patients—age variable.

	Age	N	MEAN	Std. Deviation	Std. Error Mean
SD	50–60	6	37.8667	5.18716	2.11765
	40–50	6	28.4833	5.92973	2.42080
TT	50–60	6	20.8167	8.00185	3.26674
	40–50	6	19.4333	6.78636	2.77052
PP	50–60	6	59,088.3666	20,674.8706	8440.4805
	40–50	6	36,303.8500	28,691.5870	11,713.2913

**Table 4:** Independent Samples Test—PD patients—age.

		Levene’s Test		T-Test for Equality of Means						
		for Equality								
		of Variances		95% Confidence Interval of the Difference						
							**Mean**	**Std. Error**		
		**F**	**Siq.**	**t**	**df**	**Sig.** ^1^	**Difference**	**Difference**	**Lower**	**Upper**
SD	Equal variances assumed	0.116	0.740	2.917	10.00	0.015	9.3833	3.2163	2.2169	16.5497
	Equal variances not assumed			2.917	9.83	0.016	9.3833	3.2163	2.1997	16.5669
TT	Equal variances assumed	0.464	0.511	0.323	10.00	0.753	1.3833	4.2833	−8.1606	10.9273
	Equal variances not assumed			0.323	9.74	0.754	1.3833	4.2833	−8.1952	1.9619
PP	Equal variances assumed	1.300	0.281	1.578	10.00	0.146	22,784.5166	14,437.5519	−9384.3538	54,953.3871
	Equal variances not assumed			1.578	9.09	0.149	22,784.5166	14,437.5519	−9826.3746	55,395.4080

^1^ Sig. (2-tailed).

**Table 5:** Group Statistics—PD patients—sex variable.

	Sex	N	MEAN	Std. Deviation	Std. Error Mean
SD	FEMALE	4	35.2750	3.51888	1.75944
	MALE	8	32.1250	8.54296	3.02039
TT	FEMALE	4	18.3250	7.72933	3.86466
	MALE	8	21.0250	7.14638	2.52663
PP	FEMALE	4	45,035.0500	27,235.2282	13,617.6141
	MALE	8	49,026.6375	28,139.7772	9948.9136

**Table 6:** Independent Samples Test—PD patients—sex.

		Levene’s Test		T-Test for Equality of Means						
		for Equality								
		of Variances		95% Confidence Interval of the Difference						
							**Mean**	**Std. Error**		
		**F**	**Siq.**	**t**	**df**	**Sig.** ^1^	**Difference**	**Difference**	**Lower**	**Upper**
SD	Equal variances assumed	2.598	0.138	0.69	10.00	0.50	3.150	4.533	−6.951	13.251
	Equal variances not assumed			0.90	9.90	0.39	3.150	3.495	−4.649	10.949
TT	Equal variances assumed	0.063	0.807	−0.60	10.00	0.56	2.700	4.486	−12.696	7.296
	Equal variances not assumed			−0.58	5.67	0.58	2.700	4.617	−14.160	8.760
PP	Equal variances assumed	0.068	0.799	−0.23	10.00	0.82	−3991.587	17,067.735	−42,020.872	34,037.697
	Equal variances not assumed			−0.23	6.29	0.82	−3991.587	16,864.765	−44,802.306	36,819.131

^1^ Sig. (2-tailed).

**Table 7:** Group Statistics—Healthy individuals—age variable.

	Age	N	MEAN	Std. Deviation	Std. Error Mean
SB	50–60	6	30.0000	4.81664	1.96638
	40–50	6	29.6667	6.25033	2.55169
TT	50–60	6	12.7167	3.63891	1.48558
	40–50	6	12.7333	6.71913	2.74307
PP	50–60	6	0.0194	0.00919	0.00375
	40–50	6	0.0187	0.00932	0.00381

**Table 8:** Independent Samples Test—Healthy individuals—age.

		Levene’s Test		T-Test for Equality of Means						
		for Equality								
		of Variances		95% Confidence Interval of the Difference						
							**Mean**	**Std. Error**		
		**F**	**Siq.**	**t**	**df**	**Sig.** ^1^	**Difference**	**Difference**	**Lower**	**Upper**
SD	Equal variances assumed	1.321	0.277	0.103	10.00	0.920	0.3333	3.2214	−6.8445	7.5111
	Equal variances not assumed			0.103	9.39	0.920	0.3333	3.2214	−6.9082	7.5748
TT	Equal variances assumed	1.716	0.219	−0.005	10.00	0.996	−0.0166	3.1195	−6.9673	6.9340
	Equal variances not assumed			−0.005	7.70	0.996	−0.0166	3.1195	−7.2592	7.2259
PP	Equal variances assumed	0.174	0.686	0.139	10.00	0.892	0.0007	0.0053	−0.0111	0.0126
	Equal variances not assumed			0.139	9.99	0.892	0.0007	0.0053	−0.0111	0.0126

^1^ Sig. (2-tailed).

**Table 9:** Group Statistics—Healthy individuals—sex variable.

	Sex	N	MEAN	Std. Deviation	Std. Error Mean
SD	FEMALE	6	30.5000	4.03733	1.64823
	MALE	6	29.1667	6.70572	2.73760
TT	FEMALE	6	13.2000	4.85551	1.98225
	MALE	6	12.2500	5.85414	2.38994
PP	FEMALE	6	0.0215	0.01024	0.00418
	MALE	6	0.0167	0.00728	0.00297

**Table 10:** Independent Samples Test—Healthy individuals—sex.

		Levene’s Test		T-Test for Equality of Means						
		for Equality								
		of Variances		95% Confidence Interval of the Difference						
							**Mean**	**Std. Error**		
		**F**	**Siq.**	**t**	**df**	**Sig.** ^1^	**Difference**	**Difference**	**Lower**	**Upper**
SD	Equal variances assumed	2.526	0.143	0.417	10.00	0.685	1.3333	3.1954	−5.7866	8.4533
	Equal variances not assumed			0.417	8.204	0.687	1.3333	3.1954	−6.0037	8.6703
TT	Equal variances assumed	0.096	0.763	0.306	10.00	0.766	0.9500	3.1050	−6.9673	7.8684
	Equal variances not assumed			0.306	9.669	0.766	0.9500	3.1050	−6.0006	7.9006
PP	Equal variances assumed	1.080	0.323	0.937	10.00	0.371	0.0048	0.0051	−0.0066	0.0162
	Equal variances not assumed			0.937	9.027	0.373	0.0048	0.0051	−0.0067	0.0164

^1^ Sig. (2-tailed).

## Data Availability

Not applicable.
